# Nef Alleles from All Major HIV-1 Clades Activate Src-Family Kinases and Enhance HIV-1 Replication in an Inhibitor-Sensitive Manner

**DOI:** 10.1371/journal.pone.0032561

**Published:** 2012-02-29

**Authors:** Purushottam S. Narute, Thomas E. Smithgall

**Affiliations:** 1 Department of Infectious Disease and Microbiology, Graduate School of Public Health, University of Pittsburgh, Pittsburgh, Pennsylvania, United States of America; 2 Department of Microbiology and Molecular Genetics, School of Medicine, University of Pittsburgh, Pittsburgh, Pennsylvania, United States of America; Institut Pasteur, France

## Abstract

The HIV-1 accessory factor Nef is essential for high-titer viral replication and AIDS progression. Nef function requires interaction with many host cell proteins, including specific members of the Src kinase family. Here we explored whether Src-family kinase activation is a conserved property of Nef alleles from a wide range of primary HIV-1 isolates and their sensitivity to selective pharmacological inhibitors. Representative Nef proteins from the major HIV-1 subtypes A1, A2, B, C, F1, F2, G, H, J and K strongly activated Hck and Lyn as well as c-Src to a lesser extent, demonstrating for the first time that Src-family kinase activation is a highly conserved property of primary M-group HIV-1 Nef isolates. Recently, we identified 4-amino substituted diphenylfuropyrimidines (DFPs) that selectively inhibit Nef-dependent activation of Src-family kinases as well as HIV replication. To determine whether DFP compounds exhibit broad-spectrum Nef-dependent antiretroviral activity against HIV-1, we first constructed chimeric forms of the HIV-1 strain NL4-3 expressing each of the primary Nef alleles. The infectivity and replication of these Nef chimeras was indistinguishable from that of wild-type virus in two distinct cell lines (U87MG astroglial cells and CEM-T4 lymphoblasts). Importantly, the 4-aminopropanol and 4-aminobutanol derivatives of DFP potently inhibited the replication of all chimeric forms of HIV-1 in both U87MG and CEM-T4 cells in a Nef-dependent manner. The antiretroviral effects of these compounds correlated with inhibition of Nef-dependent activation of endogenous Src-family kinases in the HIV-infected cells. Our results demonstrate that the activation of Hck, Lyn and c-Src by Nef is highly conserved among all major clades of HIV-1 and that selective targeting of this pathway uniformly inhibits HIV-1 replication.

## Introduction

The HIV-1 *nef* gene encodes a small myristoylated protein expressed early in the HIV-1 life cycle [1]–[3]. Nef, one of several accessory factors unique to primate lentiviruses, is not required for HIV-1 replication *in vitro* [4] but is essential for high-titer virus replication [5]–[9] and AIDS pathogenesis *in vivo* [10]–[13]. Nef has no known enzymatic activity, and appears to exert its effects by interacting with a diverse group of host cell proteins involved in cellular activation, immune recognition and survival [14], [15]. Many protein-protein interaction motifs have been mapped to the Nef structure, which account for its diverse functions including down-regulation of CD4 and MHC-1 molecules and activation of cellular signaling pathways [9], [16]–[37]. The essential role for Nef in HIV pathogenesis is highlighted by the requirement for this accessory factor in SIV-induced AIDS in non-human primates, and the observation that Nef-defective forms of HIV-1 have been detected in some long-term non-progressors who are seropositive but fail to develop AIDS [10], [38]. Furthermore, the CD4 promoter-directed expression of Nef alone to HIV target cells in transgenic mice is sufficient for the development of an AIDS-like syndrome characterized by CD4^+^ T cell depletion, diarrhea, wasting, and 100% mortality [39], supporting a major role for Nef in HIV-1 pathogenicity.

One important group of Nef-interacting proteins includes members of the Src protein-tyrosine kinase family [40]. Nef directly binds and activates a subset of Src-family kinases (SFKs), namely Hck, Lyn and c-Src [41]–[44]. Of these, Hck is strongly expressed in macrophages and dendritic cells, while c-Src and Lyn exhibit broader expression patterns including all HIV target cell types. Suppression of Hck expression with antisense oligonucleotides dramatically inhibits macrophage-tropic HIV-1 replication in primary human macrophages [45]. In brain-derived microglial cells, HIV-1 infection induces Nef-dependent Hck activation; suppression of Nef-Hck activity via expression of dominant-negative Hck or CD45 phosphatase inhibits HIV replication [46]. In addition, Nef-induced AIDS-like disease is delayed in Hck-null mice and completely reversed in mice expressing a Nef mutant unable to bind to Hck [39], [47]. Taken together, these results underscore the importance of Nef-SFK interaction in HIV replication and AIDS pathogenesis.

Nef interacts with SFKs by binding to their SH3 domains. Structural studies of the Nef-SH3 interface reveal that the interaction involves a highly conserved PxxPxR motif on Nef as well as a hydrophobic pocket formed by the three-dimensional fold of the Nef core region [48], [49]. Subsequent studies revealed that Nef binding induces constitutive activation of Hck and other SFKs by displacing the SH3 domain from its negative regulatory interaction with the SH2-kinase linker on the back of the kinase domain [41]–[43]. Activation requires both the Nef PxxPxR motif and hydrophobic pocket, and may induce a unique activated conformation of the partner kinase [43], [50], [51]. In addition to these highly conserved Nef regions directly involved in SH3 engagement, studies of Nef alleles derived from long-term non-progressors have shown that residues at a distance from the SH3 binding site also impact Nef-dependent Hck activation, possibly through an allosteric mechanism [52]. Thus sequence alignments alone do not necessarily predict the ability of primary Nef sequences to induce kinase activation.

In the first part of this study, we evaluated whether SFK SH3 domain binding and kinase activation are indeed properties common to Nef alleles representative of all major HIV-1 subtypes. We found that primary Nef proteins derived from the major clades of HIV-1 bind to the Hck SH3 domain and activate recombinant, downregulated Hck *in vitro*. All of the primary Nef proteins also activated Hck and Lyn (and c-Src to lesser extent) in cells, while producing no detectable effect on other SFKs. These results establish that SFK activation is indeed a function shared by Nef alleles derived from all major HIV-1 subtypes.

The highly conserved nature of Nef-SFK activation and its importance to HIV-1 replication and pathogenesis has raised interest in this signaling pathway as a therapeutic target. Recently, our laboratory developed a high-throughput screening assay for inhibitors of Nef-mediated Hck activation *in vitro*. This effort led to the discovery of 4-amino-substituted diphenylfuropyrimidine (DFP) compounds with low micromolar activity against both Nef-mediated Hck activation and Nef-dependent HIV-1 replication in cell culture [53]. These prior studies were conducted with two laboratory strains of HIV-1, SF2 and NL4-3, raising the question of the broader applicability of these compounds against the many allelic variants of HIV-1 Nef. Therefore, we investigated the effect of the DFP analogs on both primary Nef-mediated Hck activation as well as replication of HIV-1 NL4-3 Nef chimeras that express each primary Nef protein. Active analogs of DFP identified in our previous work potently inhibited primary Nef-mediated Hck activation *in vitro* as well as replication of all of the HIV-1 Nef chimeras. These results validate the Nef-SFK signaling axis as a viable target for development of broad-based inhibitors as a new approach to anti-retroviral therapeutics.

## Results and Discussion

**Figure 1:**
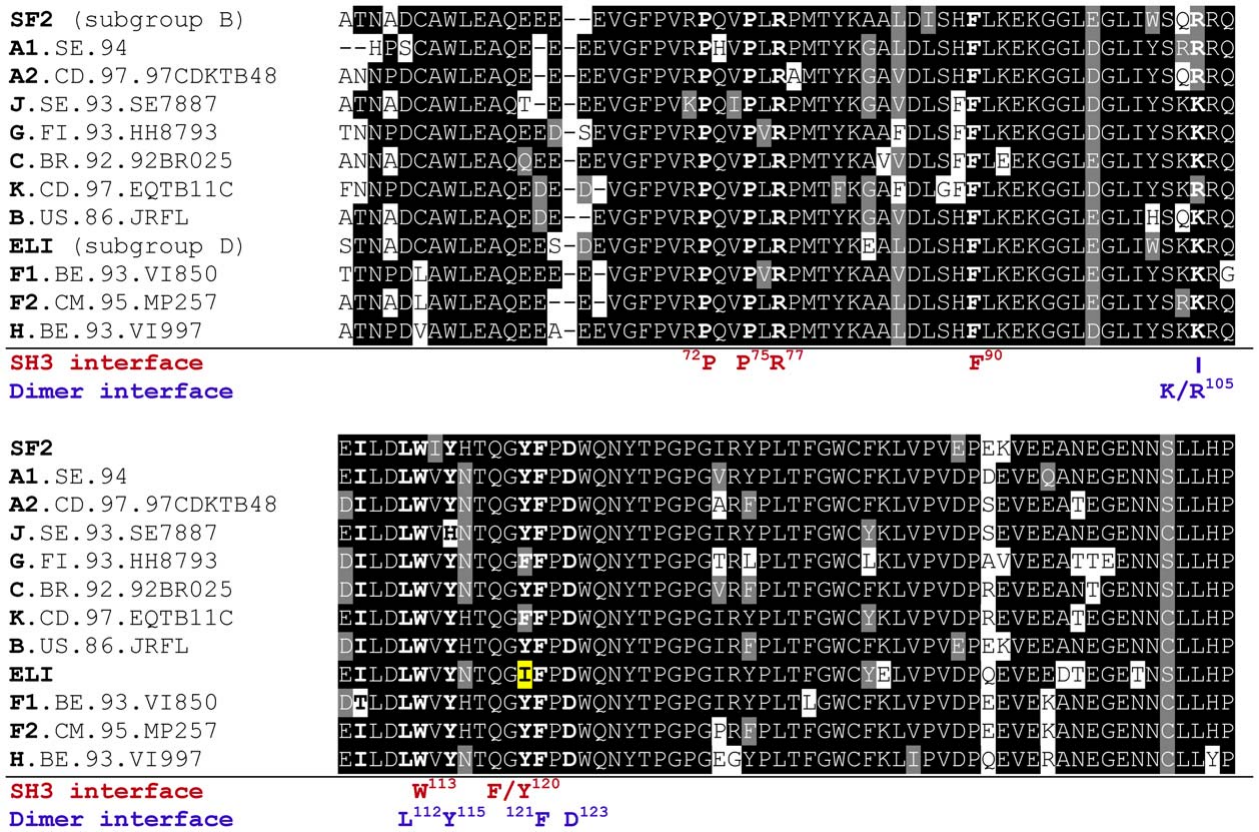
Sequence alignment of primary and laboratory Nef alleles used in this study. Nef cDNA clones representative of all major non-recombinant HIV-1 clades were selected from the NIH HIV-1 sequence database as described in the text. The first letter in each clone ID (bold) indicates the subtype assignment. This Clustal W alignment also includes Nef-SF2 (top), which has been studied extensively in our laboratory in terms of Hck activation [41], [43], [44], [50], [53], as well as Nef-ELI, which fails to activate Hck or other SFKs [44], [50]. Key residues in the SF2 sequence that are essential for SH3 binding are shown in red, and include the PxxPxR motif and hydrophobic pocket residues F90, W113, and Y/F120. In Nef-ELI, Y120 is replaced with isoleucine (highlighted in yellow); this single substitution accounts for loss of SH3 engagement and Hck activation [50]. Residues involved in Nef dimerization include K/R105, L112, Y115, F121, and D123 (blue). Flanking N- and C-terminal Nef sequences are more variable and have been omitted for clarity.

**Figure 2:**
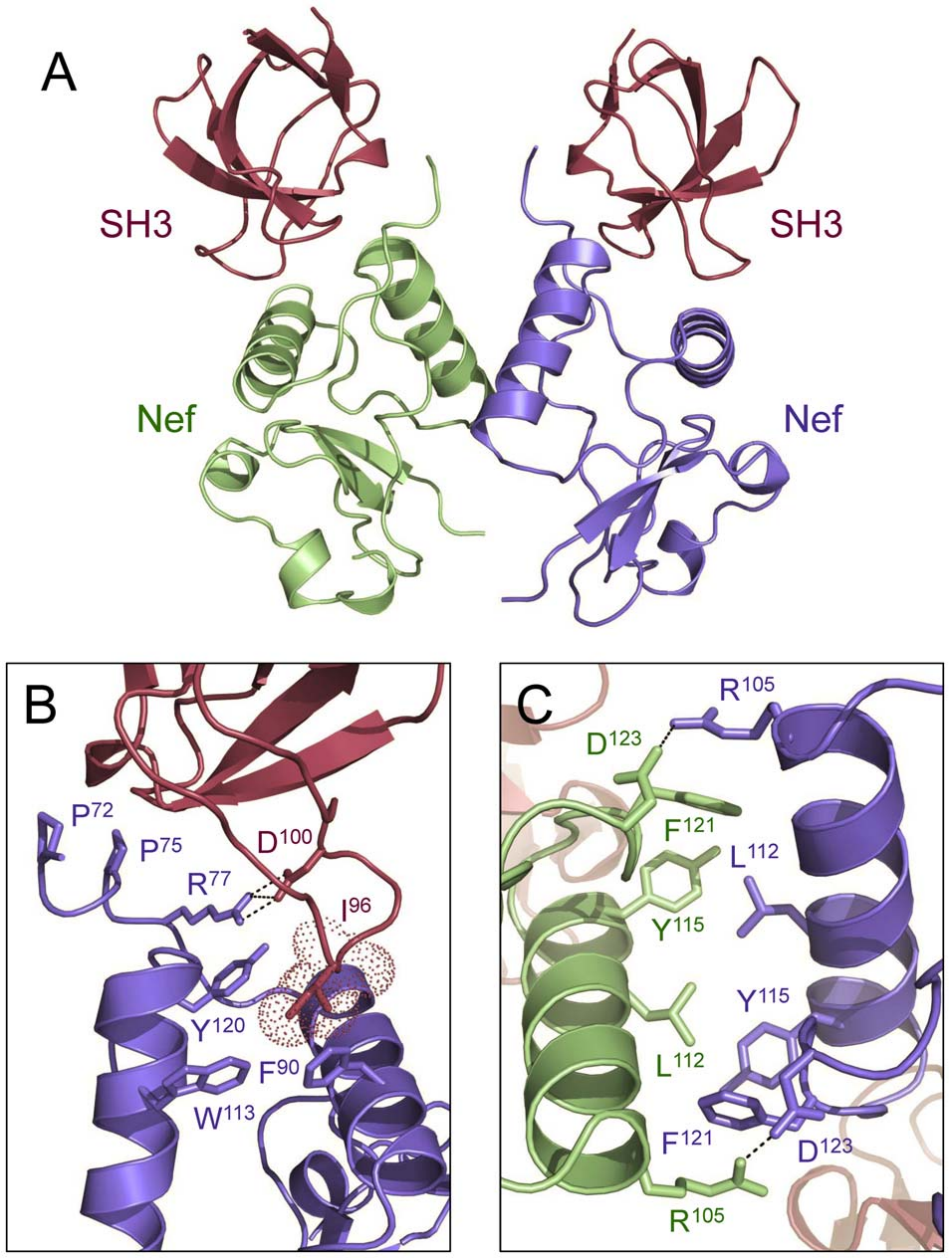
Structural features of the Nef:SH3 and Nef dimerization interfaces. A) Overview of the Nef:SH3 dimer X-ray crystal structure, based on the crystal coordinates of Lee, et al. (PDB: 1EFN) [48]. The monomeric Nef core subunits are modeled in blue and green respectively; the SH3 domains are shown in red. B) Close-up view of the Nef:SH3 interface. Nef residues P72 and P75 define a polyproline type II helix that meshes with the hydrophobic grooves of the SH3 surface, and is oriented and stabilized by an ionic interaction between Nef R77 and SH3 D100. High affinity interaction also requires a hydrophobic pocket formed in part by Nef residues F90, W113, and Y120. This pocket engages SH3 domain RT loop I96 (Van der Waals surface shown as dots). Mutagenesis of either Nef Y120 or SH3 I96 is sufficient to disrupt Nef:SH3 interaction [50], [66]. C) Close-up view of the Nef dimerization interface. Dimer packing involves hydrophobic interactions of side chains of the αB helices (L112, Y115, F121) which are orthogonally opposed. This hydrophobic core is capped on both ends by ionic interactions involving D123 and R105.

One of the major objectives of this study was to address whether SH3 domain-mediated activation of SFKs is conserved across primary Nef alleles derived from all M-group clades of HIV-1. To address this question, we assembled a set of Nef cDNA clones representative of all major non-recombinant HIV-1 subtypes. We first queried the NIH HIV-1 sequence database [54] to obtain sequences of Nef alleles from patient isolates of HIV-1 clades A1, A2, B, C, D, F1, F2, G, H, J and K. These clades are representative of the major subgroup of HIV-1 strains responsible for more than 90% of HIV-1 infections associated with the global HIV/AIDS pandemic [55]–[57]. Each of these sequences was translated *in silico* and those that encoded truncated Nef proteins were eliminated. Of the remaining sequences, one from each clade was aligned together with the laboratory alleles Nef-SF2 (clade B) and Nef-ELI (clade D). These two Nef variants have been studied extensively by our group in terms of Nef-mediated SFK activation, and serve as useful controls [43], [44], [50]. The alignment revealed strong conservation of known residues and motifs essential for SFK SH3 domain binding and kinase activation, including the PxxPxR motif and the hydrophobic pocket residues F90, W113, and Y/F120 (Figure 1). A model of the contributions of each of these residues to the Nef:SH3 interface, based on the crystal structure of Lee et al. [48], is shown in Figure 2. Note that while substitution of Y120 with isoleucine in Nef-ELI (clade D) prevents it from binding and activating Hck *in vitro* and *in vivo* [50], alignments of a larger group of primary Nef D-clade alleles revealed that this substitution is quite rare (data not shown). In addition to the SH3 domain binding site, there is also strong conservation of residues that form the Nef dimerization interface (K/R105, L112, Y115, F121, and D123). Dimerization is critical to many Nef functions, including support of HIV-1 replication and SFK activation, and mutations of the residues that make up the dimer interface in the crystal structure interfere with these Nef functions as well as Nef dimerization in a cell-based fluorescence complementation assay [58]–[60]. Figure 2 also shows a model of Nef:SH3 dimers, as well as close-up views of the Nef:SH3 and Nef:Nef interfaces.

**Figure 3:**
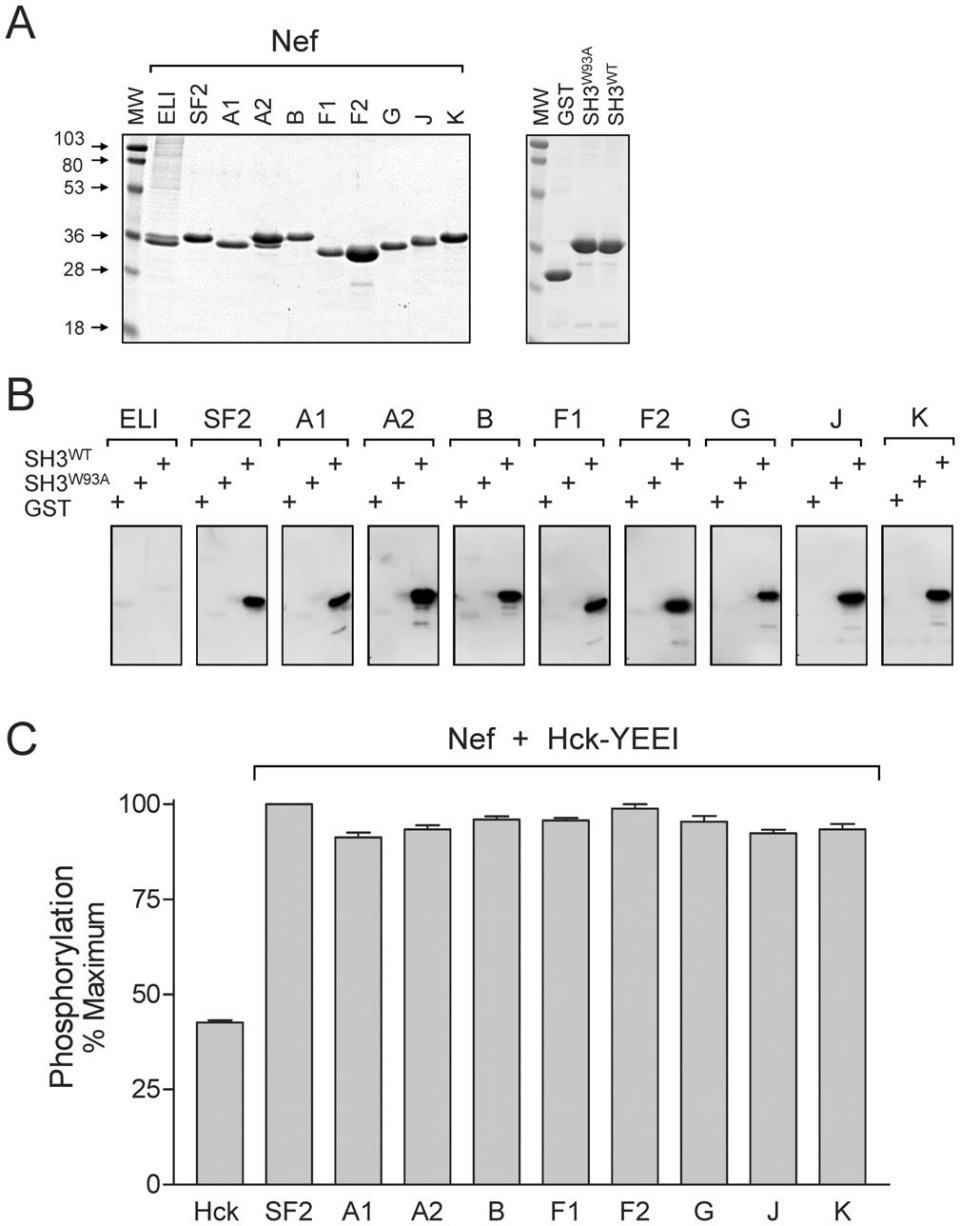
Purified primary Nef proteins bind to the Hck SH3 domain and activate downregulated Hck in vitro. A) SDS-PAGE of recombinant purified proteins. Each of the indicated Nef subtypes were expressed in bacteria and purified with N-terminal His-tags (*left*). GST and GST fusion proteins with the Hck SH3 domain (wild-type and W93A mutant) were also expressed in bacteria and immobilized on glutathione-agarose beads (*right*). Protein aliquots were resolved by SDS-PAGE and stained with Coomassie blue. B) Nef-SH3 binding assay. Recombinant His-tagged Nef proteins (1 µg) were incubated with equimolar amounts of immobilized GST and GST-SH3 fusion proteins. The agarose beads were then washed, and associated Nef proteins were detected by immunoblotting using antibodies to the His-tags. C) Nef-induced Hck activation. Kinase activity of recombinant downregulated Hck (Hck-YEEI; [44], [52], [53] was determined in the absence or presence of the indicated Nef proteins using the Z-Lyte assay as described under Materials and Methods. Results are expressed as the mean percent of maximum substrate phosphorylation ± S.D.; this experiment was repeated twice with comparable results.

To evaluate the functional properties of our M-group HIV-1 Nef clade set, we first expressed the recombinant Nef proteins in *E. coli* and purified them to homogeneity. As shown in Figure 3A, most of these primary Nef sequences yielded soluble recombinant proteins, with the exceptions of Nef-C and Nef-H. All of the recombinant Nef proteins eluted as single symmetrical peaks by gel filtration, and their molecular weights were confirmed by mass spectrometry (data not shown).

We first evaluated the binding of purified Nef proteins to the Hck SH3 domain. Because SH3 interaction requires the proper three-dimensional fold of the Nef core [48], [49], these studies provided an assessment of whether our recombinant purified Nef proteins folded correctly in bacteria. The wild-type Hck SH3 domain, as well as a binding-defective mutant (W93A; [61]), were expressed in bacteria as GST fusion proteins and purified using glutathione-agarose beads. Wild-type GST-SH3, the GST-SH3-W93A mutant control, as well as GST alone were incubated with purified recombinant Nef proteins, followed by extensive washing. Bound Nef was then visualized by immunoblot analysis. As shown in Figure 3B, all primary Nef proteins showed robust binding to the wild-type Hck SH3 domain, while no binding was observed with the W93A mutant or with GST alone. As an additional control, we also prepared recombinant Nef-ELI, the rare D-clade allele that lacks the hydrophobic pocket residue Y120 and thus fails to activate Hck [44]. As expected, Nef-ELI failed to bind to the Hck SH3 domain in this assay. Taken together, these data show that Hck SH3 domain binding is a conserved property of Nef proteins derived from M-group HIV-1 isolates.

Previous studies have established that Nef activates Hck (and other Src-family members) through displacement of the SH3 domain from its regulatory interaction with the SH2-kinase linker [42], [44]. Thus, Nef must have sufficient binding affinity to compete for the internal SH3-linker interactions present in the downregulated forms of Src-family kinases [40]. To determine whether the SH3-binding activity of our primary Nef proteins was sufficient to induce Hck activation, we turned to the FRET-based Z′-Lyte kinase assay which we have used previously to demonstrate Nef-mediated Hck activation *in vitro* (see Materials and Methods). Purified, downregulated Hck was incubated in the absence or presence of a 10-fold molar excess of each Nef protein prior to assay. As shown in Figure 3C, all of the primary Nef proteins activated Hck in the Z′-Lyte assay, demonstrating for the first time that Hck activation is a highly conserved property of M-group Nef alleles, despite considerable sequence variation.

**Figure 4:**
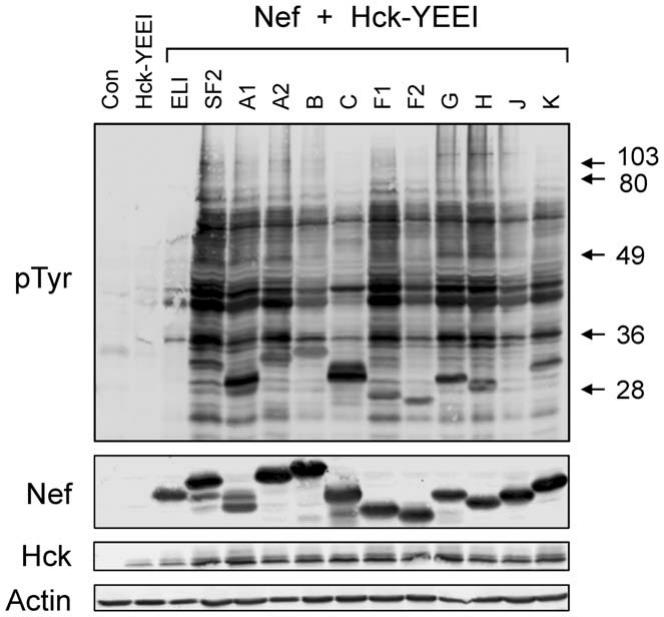
Primary M-group HIV-1 Nef proteins strongly activate Hck in cells. Yeast cultures were transformed with a galactose-inducible expression plasmid for Hck-YEEI, which carries a modified C-terminal tail that enables downregulation in the absence of Csk [52], or the empty expression plasmid as a negative control (Con). Where indicated, Hck-YEEI cells were co-transformed with galactose-inducible vectors for Nef-ELI, Nef-SF2, and ten primary Nef alleles (A1, A2, B, C, F1, F2, G, H, J, and K). Transformed cells were grown in liquid culture in the presence of galactose at 30°C for 18 h. Protein extracts were separated via SDS-PAGE and immunoblotted for tyrosine-phosphorylated proteins (*pTyr*) as well as for Nef, Hck and actin as a loading control. Nef-ELI is unable to interact with Hck and serves as an additional negative control [52]. This experiment was repeated three times with comparable results.

**Figure 5:**
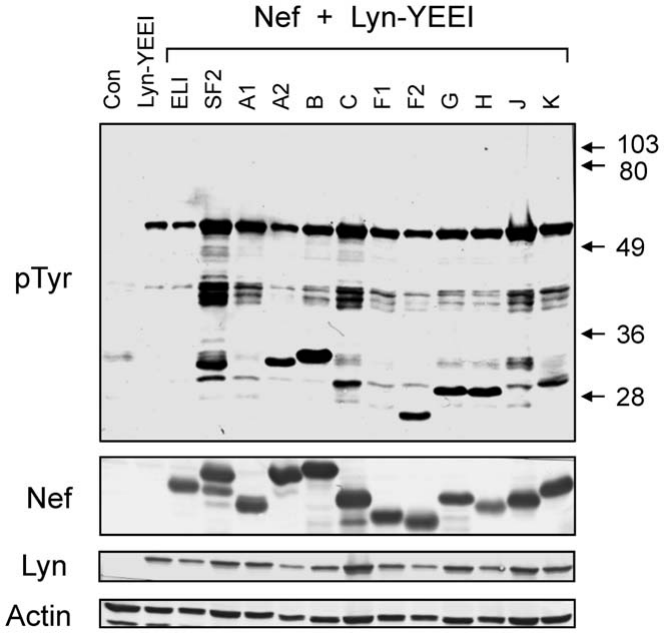
Primary M-group HIV-1 Nef proteins strongly activate Lyn in cells. Yeast cultures were transformed with a galactose-inducible expression plasmid for Lyn-YEEI, which carries a modified C-terminal tail that enables downregulation in the absence of Csk [52], or the empty expression plasmid as a negative control (Con). Where indicated, Lyn-YEEI cells were co-transformed with galactose-inducible vectors for Nef-ELI, Nef-SF2, and ten primary Nef alleles (A1, A2, B, C, F1, F2, G, H, J, and K). Transformed cells were grown in liquid culture in the presence of galactose at 30°C for 18 h. Protein extracts were separated via SDS-PAGE and immunoblotted for tyrosine-phosphorylated proteins (*pTyr*) as well as for Nef, Lyn and actin as a loading control. Nef-ELI is unable to interact with Lyn and serves as an additional negative control [52]. This experiment was repeated three times with comparable results.

Studies with recombinant Nef proteins derived from primary HIV-1 sequences all exhibited SH3-binding and Hck activation (Figure 3). However, we were not able to assess the activity of Nef-C and Nef-H, because soluble recombinant proteins could not be obtained from these sequences. To assess the interaction of these Nef proteins with Hck, and to expand our study to other members of the Src kinase family in a cell-based assay, we turned to a yeast system previously used by our group [44], [52]. In this assay, Hck and other Src-family kinases are expressed with modified “YEEI” tails so that they adopt the downregulated conformation in the absence of the negative regulatory kinase, Csk. This point is important because active Src-family kinases cause growth arrest in yeast [44]. To assess primary Nef-induced SFK activation in this system, yeast cultures were co-transformed with expression plasmids for each primary Nef sequence and Src family member, and colonies were selected on glucose agar to repress protein expression from the Gal promoter. Transformed colonies were then grown in liquid medium with galactose as sole carbon source to induce Nef and SFK expression. The cultures were lysed, and SFK activation was evaluated by immunoblotting with anti-phosphotyrosine antibodies. Nef-SF2 and Nef-ELI served as positive and negative controls, respectively. As shown in Figure 4, Nef-SF2 strongly activated Hck while Nef-ELI failed to do so, consistent with our previous data [44]. All of the primary Nef alleles tested also induced dramatic Hck activation, as reflected by the strong phosphorylation of yeast cell proteins in comparison to downregulated Hck-YEEI alone. Control experiments show that the Nef proteins do not affect yeast cell growth or protein-tyrosine phosphorylation when expressed in the absence of Hck-YEEI (Figure S1 and data not shown). Similar results were obtained with Lyn, although tyrosine phosphorylation of yeast cell proteins was less extensive for this Src-family member and may reflect more stringent substrate protein selection relative to Hck. Interestingly, Lyn also appeared to strongly phosphorylate most Nef isoforms, including A2, B, C, F2, G, H, and K, as well as SF2 (Figure 5). Variation in the extent of Nef phosphorylation by Lyn may reflect the variability in the number and position of the tyrosine residues present in individual Nef proteins. SFK-dependent tyrosine phosphorylation of Nef in HIV host cells may influence its function or interaction with other binding partners.

In addition to Hck and Lyn, we also evaluated the effect of the primary Nef alleles on other SFKs expressed in HIV-1 target cells. All of the Nef alleles induced partial activation of c-Src in this system (Figure S2), which was more apparent in growth suppression assays than the anti-phosphotyrosine immunoblots with the exceptions of Nef-F2 and Nef-H (Figure S3). In contrast, no activation of Lck, Fyn or Fgr was observed with any of the primary Nef alleles tested (Figure S4). These data demonstrate that Hck and Lyn (and c-Src to a lesser extent) are selectively activated by M-group HIV-1 Nef proteins, supporting a role for these kinases as direct Nef effectors in HIV-infected cells.

**Figure 6:**
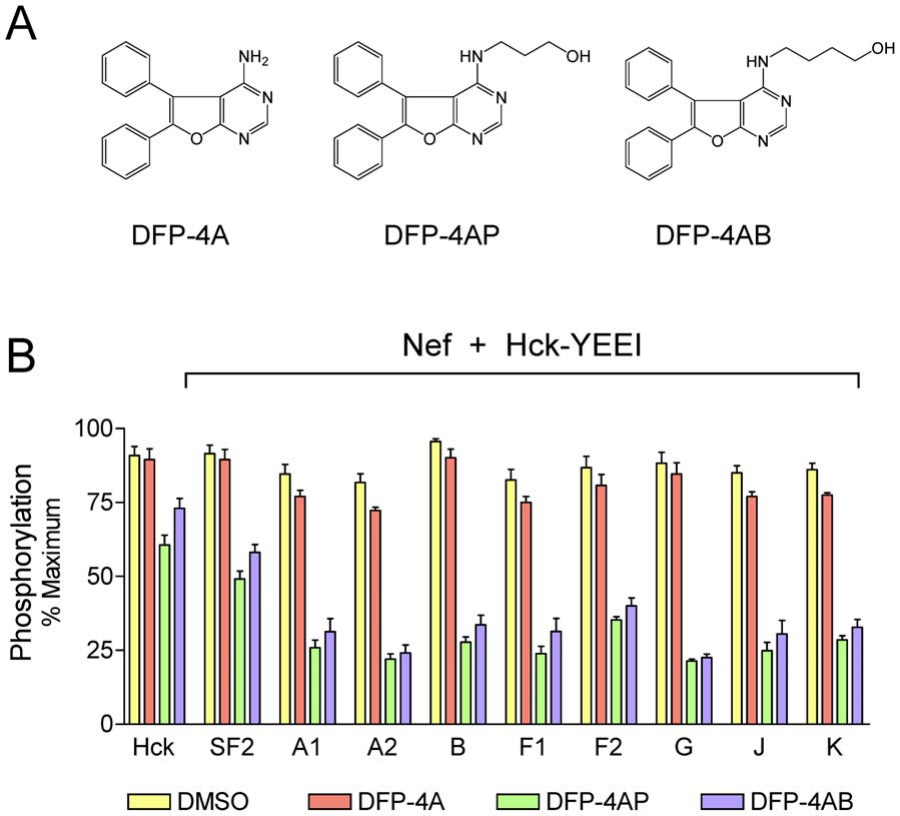
Diphenylfuropyrimidine (DFP) analogs are broadly active against primary Nef-mediated Hck activation. A) Structures of 4-amino DFP (DFP-4A) and the corresponding 4-aminopropanol (DFP-4AP) and 4-aminobutanol (DFP-4AB) analogs. B) The activity of recombinant downregulated Hck (Hck-YEEI) [44], [52], [53] was determined in the absence or presence of the indicated Nef proteins as described under Materials and Methods. Reactions were run in the presence of 10 µM concentrations of DFP-4A, DFP-4AP, DFP-4AB, or the DMSO carrier solvent as negative control. Results are expressed as the mean percent of maximum substrate phosphorylation ± S.D.; this experiment was repeated twice with comparable results.

Recently we described a screening assay for small molecule inhibitors of Nef-dependent Hck activation *in vitro* [53]. Using this assay, we identified a series of 4-amino-diphenylfuropyrimidines (DFPs) as potent inhibitors of both Nef-SF2-mediated Hck activation and Nef-dependent HIV-1 replication. Because these compounds represent potential leads for Nef-directed HIV-1/AIDS therapeutics, we next examined whether these compounds inhibit Hck following activation by each of the recombinant primary Nef proteins using the Z′-Lyte kinase assay. As shown in Figure 6, both the 4-aminopropanol and 4-aminobutanol derivatives of DFP inhibited primary Nef-induced Hck activation by more than 50% at 10 µM. Remarkably, the inhibitory action of both compounds was more pronounced with the primary Nef proteins than with Nef-SF2, the laboratory allele originally used to develop the screen. In contrast, the unsubstituted 4-amino DFP pharmacophore was without effect, consistent with our previous results [53]. These data demonstrate that 4-amino substituted derivatives of DFP are broadly active against the Hck:Nef complex independent of the Nef isolate used.

**Figure 7:**
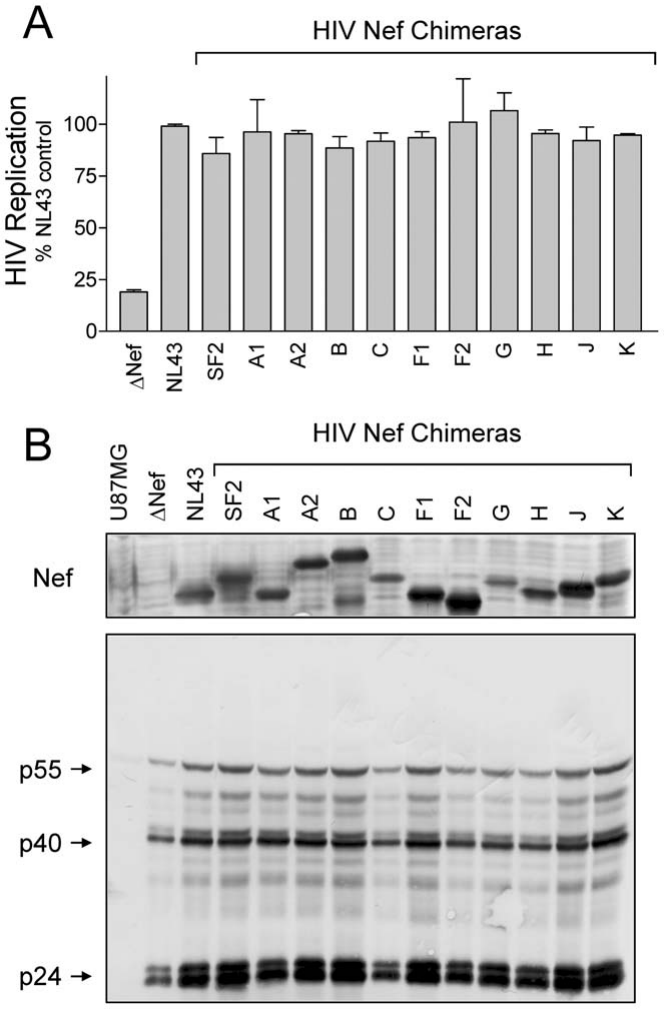
Replication of HIV-1 Nef chimeras in U87MG-CD4^+^/CXCR4^+^ cells. A) U87MG- CD4^+^/CXCR4^+^ cells (2×10^4^ per well of a 96-well plate) were infected with 200 pg p24 equivalents/ml of wild-type HIV-1 NL4-3, a Nef-defective mutant (ΔNef), or the indicated Nef chimeras in a final culture volume of 200 µl. HIV p24 levels were determined by ELISA 5 days later. Data are presented as percent of p24 release observed relative to the HIV-1 NL4-3 control ± S.D. B) U87MG-CD4^+^CXCR4^+^ cells (1×10^5^ per well of a 6-well plate) were infected with 1 ng p24 equivalents/ml of the same panel of viruses as in part A. Viral Nef and gag protein expression was verified by immunoblotting lysates of infected cells 4 days later. Lysates from uninfected cells are included as a negative control (U87MG; far left lane).

**Figure 8:**
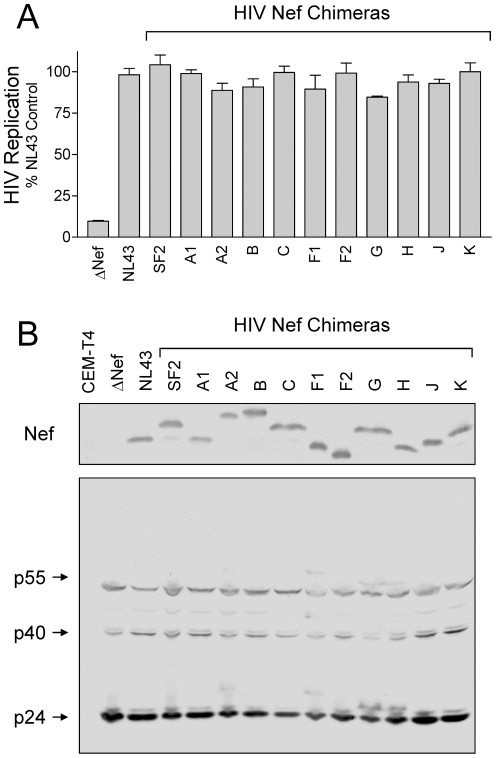
Replication of HIV-1 Nef chimeras in CEM-T4 cells. A) CEM-T4 cells (1×10^4^ per well of a 96-well plate) were infected with 62.5 pg p24 equivalents/ml of wild-type HIV-1 NL4-3, a Nef-defective mutant (ΔNef), or the indicated Nef chimeras in a final culture volume of 200 µl. HIV p24 levels were determined by ELISA 10 days later. Data are presented as percent of p24 release observed relative to the NL4-3 control ± S.D. B) CEM-T4 cells (5×10^5^ per well of a 6-well plate) were infected with 1 ng p24 equivalent/ml of the same panel of viruses as in part A. Viral Nef and gag protein expression was verified by immunoblotting lysates of infected cells 6 days later. Lysates from uninfected cells are included as a negative control (CEM-T4; far left lane).

Previous work from our group and others has established that Nef expression is essential for optimal replication of HIV-1 in the astroglioma cell line U87MG which has been engineered to express CD4 and the co-receptor CXCR4, and in the T-cell line, CEM-T4 [53], [62], [63]. Replication of HIV-1 strain NL4-3 is potently inhibited by 4-amino-substituted DFP compounds in the U87MG model system, and this inhibitory effect is dependent upon the expression of Nef [53]. In order to investigate the broader utility of the DFP compounds against M-group HIV-1 isolates, we first needed to demonstrate functional enhancement of HIV-1 replication by each of our primary Nef alleles in these cell lines. To accomplish this goal, we generated a series of HIV-1 NL4-3 chimeras in which the NL4-3 Nef sequence was replaced by each of the representative M-group Nef coding sequences. The resulting chimeric viruses were first used to infect the T cell line MT-2, where they induced syncitia formation indistinguishable from wild-type NL4-3 and replicated strongly (data not shown). We then evaluated the ability of each primary Nef allele to enhance HIV-1 replication in U87MG-CD4^+^/CXCR4^+^ cells. As observed previously, Nef-defective HIV-1 replicated very poorly in this cell line (Figure 7A). However, replication of each of the Nef chimeras was indistinguishable from wild-type HIV-1 NL4-3, demonstrating that these Nef proteins function to support HIV replication. Immunoblots (Figure 7B) demonstrate that each Nef variant is expressed in infected cells, along with major capsid proteins. Similarly, replication of Nef-defective HIV-1 replicated to only 10% of wild-type levels in CEM-T4 cells, and this replication defect was completely rescued by *cis*-complementation with all of the primary Nef alleles tested (Figure 8A). Immunoblotting verified Nef expression in CEM-T4 cells infected with wild-type HIV-1 and each of the chimeras, as well as capsid proteins (Figure 8B). These results demonstrate that HIV-1 replication is Nef-dependent in both the U87MG-CD4^+^/CXCR4^+^ and CEM T4 cell lines, providing an important new tool to assess the broad efficacy of small molecule inhibitors of Nef function on M-group HIV-1 replication.

**Figure 9:**
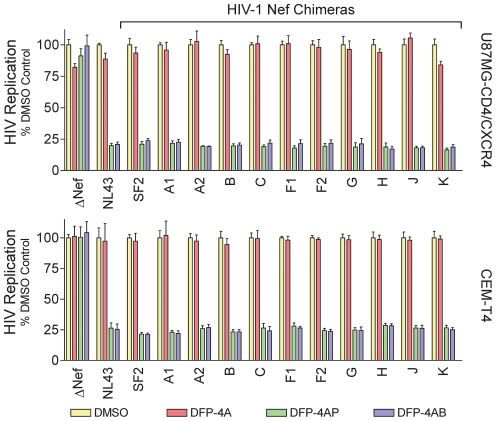
Inhibition of HIV-1 Nef chimeras by 4-amino DFP analogs. U87MG-CD4^+^/CXCR4^+^ cells (upper panel) and CEM-T4 cells (lower panel) were infected with wild-type HIV-1 NL4-3, a Nef-defective mutant (ΔNef), or the indicated Nef chimeras as described in the legends to Figures 7 and 8. DFP-4A, DFP-4AP, or DFP-4AB were added to the cultures to a final concentration of 3 µM, and viral replication was determined by p24 ELISA 5 days (U87MG) or 10 days (CEM-T4) later. Data are expressed as the mean percent of HIV-1 replication observed in control cultures incubated with the carrier solvent (0.1% DMSO) ± S.D. (n = 6).

As described in the preceding sections, 4-amino substituted DFP analogs are potent inhibitors of both Nef-dependent SFK activation and HIV replication [53]. Two DFP derivatives, DFP-4-AP and DFP-4-AB, inhibited Hck activation by all of the recombinant M-group Nef proteins tested (Figure 6), suggesting that they may inhibit replication of the HIV-1 chimeras expressing each of these primary Nef alleles as well. To test this possibility, we infected both U87MG-CD4^+^/CXCR4^+^ and CEM-T4 cells with wild-type HIV-1 NL4-3, the Nef-defective mutant and the Nef chimeras in the absence or presence of DFP-4-AP or DFP-4-AB. As shown in Figure 9, both of these DFP analogs inhibited replication of wild-type HIV-1, as well as all of the Nef chimeras, by more than 75% at a concentration of 3 µM in both cell lines. In contrast, the compounds had no impact on the replication of Nef-defective HIV-1, demonstrating that the antiretroviral activity of the DFP analogs requires the expression of Nef. As with the *in vitro* kinase assays, the unsubstituted 4-amino DFP pharmacophore was completely inactive, consistent with our original observations [53].

**Figure 10:**
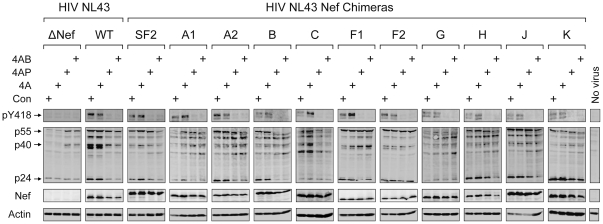
Inhibition of HIV-1 Nef chimera-mediated activation of SFKs by 4-amino DFP analogs. CEM-T4 cells were infected with wild-type HIV-1 NL4-3 (WT), a Nef-defective mutant (ΔNef), or the indicated Nef chimeras in the absence or presence of 3 µM DFP-4A, DFP-4AP, DFP-4AB, or the carrier solvent (DMSO) alone as a control (Con). SFK proteins were immunoprecipitated from infected cell lysates followed by immunoblotting with a phosphospecific antibody against the activation loop phosphotyrosine residue common to all SFKs (pY418). Control blots were performed on cell lysates for HIV-1 Gag proteins (p55, p40, and p24), Nef, as well as actin as a loading control. Results from uninfected cells are shown in the far right lane (No virus). This experiment was repeated twice with comparable results.

In a final series of experiments, we investigated the effect of HIV-1 infection on endogenous SFK activation in CEM-T4 T cells, as well as the impact of the DFP-based inhibitors on HIV-mediated kinase activation. For these studies, CEM-T4 cells were infected with wild-type HIV NL4-3, the Nef-defective mutant, as well as each of the eleven Nef chimeras. Infected cells were lysed and SFK activity was monitored with a phosphospecific antibody that recognizes the phosphotyrosine residue in the activation loop of active SFKs (pY418). As shown in Figure 10, HIV infection resulted in a Nef-dependent increase in endogenous Src-family kinase activation in every case. Immunoblots with SFK isoform-specific antibodies revealed that CEM-T4 cells express the direct Nef targets c-Src and Hck (Figure S5), which are likely to be the SFKs activated by HIV infection in this system. Furthermore, when cells were treated with DFP-4AB or DFP-4AP, both of which block Nef-dependent HIV-1 replication (Figure 9), SFK activation was inhibited. In contrast, the inactive DFP analog, DFP-4A, had no effect on kinase activity. These data provide important new evidence that HIV-1 infection results in sustained SFK activation in a Nef-dependent manner. This function is shared by Nef alleles derived from all major HIV-1 clades in the context of HIV-1 replication. Furthermore, this pathway is sensitive to the DFP compounds, strongly supporting inhibition of this pathway as their primary mechanism of action. These results support the conclusion that therapeutic targeting of Nef-dependent SFK activation may represent a broadly useful strategy against HIV-1 replication.

In summary, our study demonstrates for the first time that Src-family kinase binding and activation are highly conserved properties of Nef isolates representative of all major HIV-1 subtypes. As observed previously for Nef proteins derived from laboratory strains of HIV-1 such as SF2 [44], [50], direct SFK activation by Nef is limited to Hck and Lyn, with some activation apparent for c-Src. These observations support the formation of specific Nef-SFK complexes in all HIV target cell types. In contrast, Fyn, Lck, and Fgr, which are also expressed in HIV host cells, do not appear to be direct Nef effectors. In prior work, we identified selective inhibitors of Nef-SF2-mediated Hck activation *in vitro* that also blocked enhancement of HIV-1 replication by Nef [53]. In the present study, we demonstrate that these 4-amino substituted DFP analogs exhibit a broad spectrum of activity against Nef:Hck complexes *in vitro*. Importantly, the DFP-based compounds show equipotent inhibition of Nef-dependent HIV-1 replication against a panel of HIV-1 Nef chimeras representative of all major HIV-1 clades. These observations strongly support the development of 4-amino DFP analogs as a first step towards new antiretroviral agents that target the interaction of this key HIV-1 virulence factor with host cell signaling proteins.

## Materials and Methods

### Expression vectors

Nucleotide sequences encoding representative Nef proteins from the HIV-1 M-group subtypes A1, A2, B, C, F1, F2, G, H, J and K were synthesized commercially (DNA2.0). The Nef sequences were PCR-amplified and subcloned into the bacterial expression vector pET14b (Novagen/EMD Biosciences). Construction of similar bacterial expression vectors for Nef-SF2 and Nef-ELI has been described [52]. For yeast studies, the Nef sequences from the DNA2.0 plasmids were subcloned directly into the yeast expression vectors pESC-Trp or pESC-Trp-Csk under the control of the GAL10 promoter as described previously [44]. Similar pESC and pYC2/CT-based vectors for yeast expression of Hck, Lyn, Lck, Fyn, Fgr, and c-Src as well as the *Herpesvirus saimiri* Tip protein have been reported previously by our group [44], [52], [64].

### Cell lines and antibodies

Human 293T cells were obtained from the ATCC and grown in Dulbecco's Modified Eagle's Medium/high glucose (Life Technologies) supplemented with 10% fetal bovine serum (Gemini Bio-Products). U87MG-CD4^+^/CXCR4^+^, MT2 and CEM-T4 cells were obtained from NIH AIDS Research and Reference Reagent Program. U87MG-CD4^+^/CXCR4^+^ cells were grown in Dulbecco's Modified Eagle's Medium/high glucose supplemented with 25 mM HEPES, pH 7.4, 10% fetal bovine serum, G418 (400 µg/ml) and puromycin (0.5 µg/ml); G418 and puromycin are required to maintain expression of CD4 and CXCR4 in this cell line. MT2 and CEM-T4 cells were grown in RPMI 1640 medium (Life Technologies) supplemented with 10% fetal bovine serum and 2 mM L-glutamine.

Antibodies used in this study were obtained from the NIH AIDS Research and Reference Reagent Program (Nef, 2949; p24, 4121), Santa Cruz Biotechnology (pY99, sc-7020; Hck, sc-72; Lyn, sc-15; Fyn, sc-16; Lck, sc-13; c-Src, sc-18; Csk, sc-286; Fgr, sc-17; pan-specific SFK, sc-5266; His-tag, sc-803; GST, sc-138), Abcam (c-Yes, Ab13954), Life Technologies (SFK pY418, 44660G) and Millipore (actin, MAB 1501).

### Nef and Hck protein purification

*E. coli* strains BL21 DE3 Rosetta2 (Novagen) or Arctic Express (Stratagene) were transformed with pET14b-Nef plasmids and protein expression was induced with 0.5 mM IPTG at 28°C for 4 h (Rosetta2) or 11°C for 24 h (Arctic Express). The bacterial cells were collected by centrifugation and lysed in His-lysis buffer (20 mM Tris-HCl, pH 8.3, 500 mM NaCl, 20 mM imidazole, 10% glycerol and 5 mM β-mercaptoethanol). Lysates were clarified by centrifugation at 30,000×g at 4°C for 30 min. The His-tagged Nef proteins were purified by Ni-NTA affinity chromatography using an ÄKTAexplorer automated chromatography system (GE Healthcare). Lysates containing His-tagged Nef were loaded on a Ni-NTA HiTrap Chelating HP column (5 ml; GE Healthcare) pre-equilibrated with lysis buffer. After washing with 2 column volumes (CV) of lysis buffer, proteins were eluted in His-elution buffer (20 mM Tris-HCl, pH 8.3, 1 M imidazole, 10% glycerol and 5 mM β-mercaptoethanol) with a linear gradient of 20 mM to 1 M imidazole over 6 CV. The His-tagged Nef proteins were further purified by gel filtration chromatography on a HiLoad 26/60 Superdex 75 column (GE Healthcare) with 20 mM Tris-HCl, pH 8.3, 100 mM NaCl and 3 mM DTT as mobile phase. Recombinant near full-length Hck was purified in its downregulated conformation (Hck-YEEI) from Sf-9 insect cells as described in detail elsewhere [44], [53].

### Nef-SH3 domain binding assay

GST-tagged Hck SH3 domain fusion proteins (wild-type and W93A mutant) as well as GST alone were expressed in *E. coli* BL21 Rosetta cells and immobilized on glutathione-agarose beads according to our published protocol [61]. For Nef-SH3 binding assays, each recombinant purified Nef protein (1 µg) was incubated with an equimolar amount of immobilized GST or the GST-SH3 fusion proteins in 500 µl of lysis buffer (50 mM Tris-HCl, pH 7.4, 50 mM NaCl, 10 mM Mg_2_Cl, 1 mM EDTA, 1% Triton X-100) supplemented with protease inhibitors at 4°C for 2 h. Binding reactions were then washed by resuspending the beads 4 times in 1 ml RIPA buffer (50 mM Tris-HCl, pH 7.4, 150 mM NaCl, 1 mM EDTA, 1% Triton X-100, 0.1% SDS, 1% sodium deoxycholate) supplemented with protease inhibitors. Associated Nef proteins were eluted in SDS-PAGE sample buffer at 95°C for 10 min, separated by SDS-PAGE, and visualized by immunoblotting with antibodies against the N-terminal His tag.

### In vitro kinase assay

Nef-mediated activation of Hck tyrosine kinase activity was assayed using the Z′-Lyte method with the Tyr2 peptide substrate (Life Technologies). The principle of this FRET-based assay is described in detail elsewhere [44], [53]. Briefly, reactions (10 µl) were conducted in 384-well plates. Recombinant Hck (25 ng) was incubated with a 10-fold molar excess of each recombinant Nef protein in the absence or presence of DFP-4A, DFP-4-AP, or DFP-4AB (10 µM) [53] in kinase assay buffer (50 mM Hepes, pH 7.5, 10 mM MgCl_2_, and 1 mM EGTA, 0.01% Brij-35) for 1 h at room temperature. For experiments with Hck in the absence of Nef, higher input kinase was used (75 ng) to achieve equivalent levels of substrate phosphorylation to that observed in the presence of Nef. Development reagent was then added to the reaction for an additional 60 min at room temperature, followed by the stop reagent. Fluorescence was assessed at an excitation wavelength of 400 nm; coumarin fluorescence and the fluorescein FRET signal were monitored at 445 and 520 nm, respectively. Reactions run in the absence of ATP served as the 0% phosphorylation control, whereas stoichiometrically phosphorylated Tyr2 peptide was used as the 100% phosphorylation control. Raw fluorescence values were corrected for background, and reaction endpoints calculated as emission ratios of coumarin fluorescence divided by the fluorescein FRET signal. These ratios were then normalized to the ratio obtained with the 100% phosphorylation control peptide. Each condition was assayed in quadruplicate, and results are presented as the mean ± S.D.

### Yeast assay for SFK activation

The yeast-based assay for analysis of SFK activation by Nef and other viral proteins is described in detail elsewhere [44], [52], [64]. Briefly, *Saccharomyces cerevisiae* strain YPH 499 (Stratagene) was co-transformed with pESC-Ura (or pYC2/CT-Ura) and pESC-Trp plasmids containing the genes of interest via electroporation (Bio-Rad GenePulser II). Transformed colonies were selected on synthetic drop-out medium lacking uracil and tryptophan with glucose as sole carbon source for 3 d at 30°C. Colonies were cultured in galactose-containing medium lacking uracil and tryptophan for 18 h at 30°C to induce gene expression. Cell densities were normalized to an OD_600_ of 0.2 in water and lysed with 0.1 N NaOH for 5 min at room temperature. Aliquots of each lysate were separated via SDS-PAGE, transferred to polyvinylidene difluoride (PVDF) membranes, and probed for protein phosphotyrosine content by immunoblotting with anti-phosphotyrosine antibodies. Replicate blots were probed for expression of each SFK, Nef and actin as a loading control.

### HIV-1 Nef chimera construction and viral replication assays

To generate the HIV-1 Nef chimeras, unique *Cla* I restriction sites flanking the Nef ORF were introduced into pUC18 carrying the complete HIV-1 NL4-3 proviral DNA sequence (pUC18-NL4-3) [53], [58]. The coding sequences for the primary Nef genes as well as Nef-SF2 were PCR-amplified with *Cla* I linkers and used to replace the NL4-3 Nef sequence in pUC18-NL4-3. Control and chimeric viruses were generated by transfection of 293T cells, followed by amplification of the viral supernatants in the T cell line, MT2. For HIV-1 replication assays, U87MG-CD4^+^/CXCR4^+^ and CEM-T4 cells were incubated overnight in the absence or presence of the DFP analogs in a final concentration of 0.1% DMSO as carrier solvent. U87MG-CD4^+^/CXCR4^+^ cells were then infected with 200 pg p24/ml of each chimeric virus and incubated for 5 d while CEM-T4 cells were infected with 62.5 pg p24/ml of each chimeric virus for 10 d in 96-well plates. HIV-1 levels in the culture supernatants were then determined by p24 ELISA as described [53].

### Activation of endogenous SFKs by HIV-1 Nef chimeras

CEM-T4 cells (1×10^5^ per T25 flask) were infected with 50 pg p24 equivalents/ml of wild-type HIV-1 NL4-3, a Nef-defective mutant (ΔNef), or the indicated Nef chimeras in a final culture volume of 10 ml in the absence or presence of 3 µM DFP-4A, DFP-4AP, DFP-4AB, or the carrier solvent (DMSO) alone as a control (Con). The infected cells were lysed eight days later in RIPA buffer and SFK proteins were immunoprecipitated with a pan-specific antibody and protein-G sepharose beads as described elsewhere [50], [65]. SFK activation was assessed by immunoblotting each immunoprecipitate with a phosphospecific antibody against the activation loop phosphotyrosine residue common to all Src family members (pY418; [61]). Control blots were performed on cell lysates for HIV-1 Gag proteins (p55, p40, and p24), Nef, as well as actin as a loading control.

## Supporting Information

Figure S1 **Expression of Nef alone, or together with the SFK regulator Csk, has no effect on tyrosine phosphorylation of yeast proteins.** Control experiments investigated whether Nef alone (or co-expression of Nef with the SFK regulator, Csk) had any effect on tyrosine phosphorylation of yeast proteins. Yeast cultures expressing each primary Nef allele as well as Nef-SF2 and Nef-ELI were grown in defined medium with galactose as sole carbon source to induce gene expression. Yeast protein-tyrosine phosphorylation was evaluated by immunoblotting of yeast cell lysates. None of the Nef proteins induced tyrosine phosphorylation in the absence of a co-expressed SFK. Co-expression of Nef-SF2 with Hck is included as a positive control (left panels). Co-expression of Nef with Csk, the negative regulator of SFKs, also did not induce yeast protein-tyrosine phosphorylation (right panels). In contrast, expression of c-Src (in the absence of Csk) led to strong tyrosine phosphorylation of yeast proteins. These results indicate that expression of Nef alone or Nef with Csk does not induce tyrosine phosphorylation of yeast proteins, consistent with the lack of endogenous SFK orthologs in yeast.
(TIFF)

Figure S2 **Primary HIV-1 Nef proteins induce modest activation of c-Src in yeast.** Unlike the other SFKs used in the yeast studies, modification of the c-Src tail with a YEEI tail did not result in effective downregulation in the absence of Csk. Therefore we decided to downregulate wild-type c-Src by co-expression of Csk as described previously. Yeast cultures were co-transformed with c-Src, Csk, and Nef expression plasmids, and protein expression was induced in galactose medium as described under Materials and Methods. Protein-tyrosine phosphorylation was then evaluated by anti-phosphotyrosine immunoblotting. Expression of c-Src alone induced strong phosphorylation of yeast proteins that was downregulated in the presence of Csk (Src+Csk). Co-expression of Nef-SF2 overcame Csk-mediated c-Src inhibition, while Nef-ELI failed to activate c-Src. All of the primary Nef proteins also activated Csk-downregulated c-Src kinase, albeit to a lesser extent compared to Nef-SF2.
(TIFF)

Figure S3 **Co-expression of c-Src with primary Nef proteins induces growth arrest in yeast.** To look for additional evidence of Nef-mediated c-Src activation, we also monitored yeast cell growth. Our previous work has shown that ectopic expression of active SFKs induces growth arrest in yeast, providing another measure of Nef-SFK interaction in this system. For these experiments, aliquots of the transformed yeast cultures shown were spotted over a series of dilutions on galactose-agar plates, and the yeast patches appear as dark circles in the resulting scanned images of the plates. Expression of c-Src alone induced growth suppression compared to control cultures, while expression of c-Src with Csk reversed this effect. Consistent with our previous work, expression of Nef-SF2 overcame the inhibition of c-Src by Csk, leading to growth suppression, while the non-interacting allele Nef-ELI failed to do so. Co-expression of primary Nef proteins with c-Src also induced growth suppression in the presence of Csk, with the exceptions of Nef-A2, Nef-F2 and Nef-H. These data provide additional evidence that primary Nef proteins from almost all major HIV-1 clades activate c-Src. To control for culture plating density, replicate dilutions of each culture were also spotted on glucose-agar plates. Because glucose represses protein expression from the GAL promoter, c-Src, Csk, and Nef are not expressed and all cultures grow to the same extent.
(TIFF)

Figure S4 **Primary HIV-1 Nef proteins do not activate Lck, Fyn or Fgr in yeast.** Here we examined whether primary Nef proteins can activate other SFKs expressed in HIV-1 target cells using the yeast assay. Primary Nef proteins were co-expressed with the down-regulated (YEEI) forms of Lck, Fyn and Fgr, followed by anti-phosphotyrosine immunoblotting of yeast cell lysates. Co-expression with Nef had no effect on Lck, Fyn or Fgr kinase activity despite strong expression of the Nef proteins and each SFK. As a positive control, we co-expressed Lck-YEEI with the herpesvirus Tip protein, which binds to Lck and induces strong kinase activation. Fyn and Fgr were also expressed as their wild-type forms; in the absence of Csk, these SFKs induced strong yeast tyrosine phosphorylation.
(TIFF)

Figure S5 **Src-family kinase expression in CEM-T4 lymphoblasts.** CEM-T4 cells were lysed in RIPA buffer (see main text) and SFK protein expression was assessed by immunoblotting with antibodies specific for the individual Src family members shown. Lysates were also blotted with actin antibodies as a loading control.
(TIFF)
