# Supplementary figures and images for: Nef Alleles from All Major HIV-1 Clades Activate Src-Family Kinases and Enhance HIV-1 Replication in an Inhibitor-Sensitive Manner
Source: PLoS One. 2012 Feb 29;7(2):e32561. doi: 10.1371/journal.pone.0032561 (PMC3290594; doi:10.1371/journal.pone.0032561)

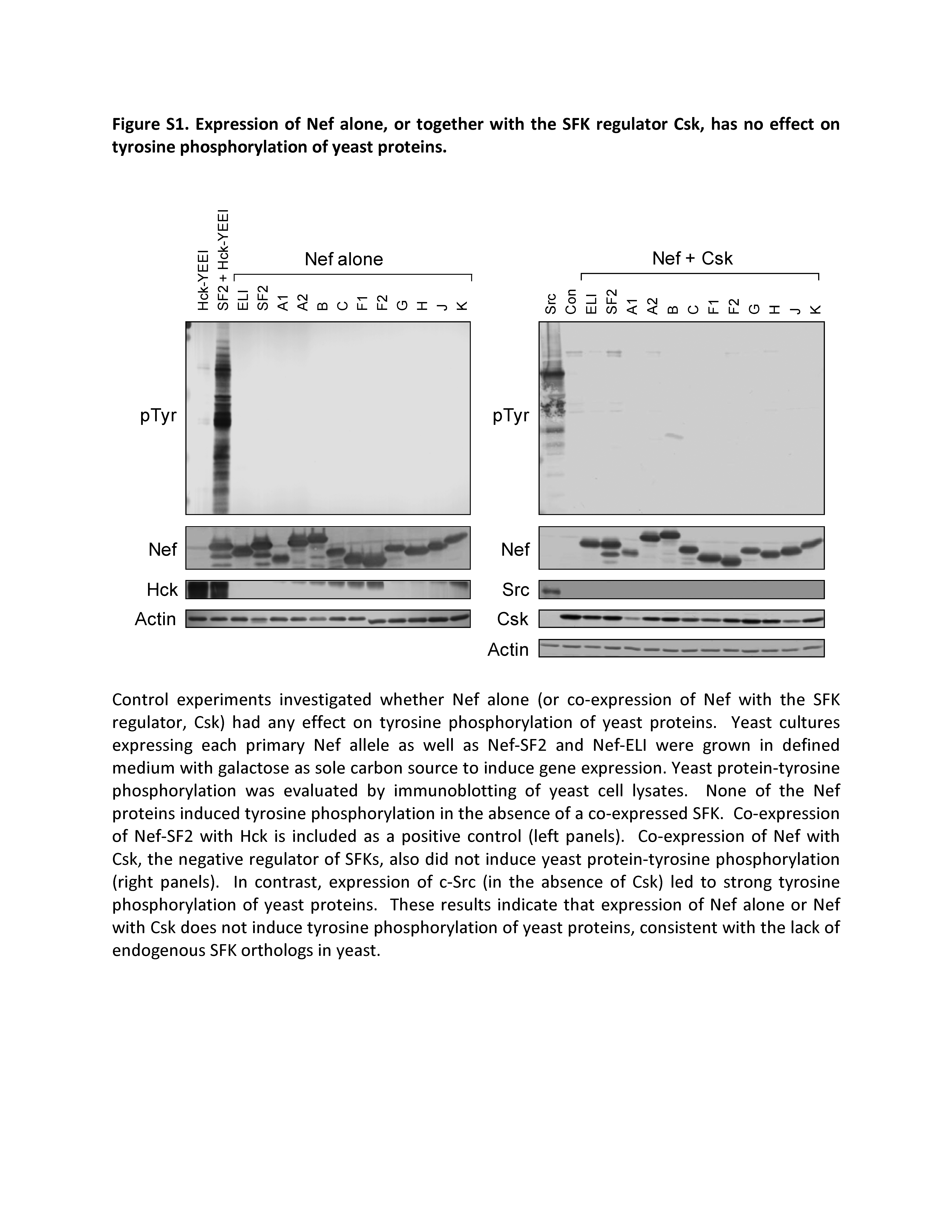

Supplement: Figure S1 — Expression of Nef alone, or together with the SFK regulator Csk, has no effect on tyrosine phosphorylation of yeast proteins. Control experiments investigated whether Nef alone (or co-expression of Nef with the SFK regulator, Csk) had any effect on tyrosine phosphorylation of yeast proteins. Yeast cultures expressing each primary Nef allele as well as Nef-SF2 and Nef-ELI were grown in defined medium with galactose as sole carbon source to induce gene expression. Yeast protein-tyrosine phosphorylation was evaluated by immunoblotting of yeast cell lysates. None of the Nef proteins induced tyrosine phosphorylation in the absence of a co-expressed SFK. Co-expression of Nef-SF2 with Hck is included as a positive control (left panels). Co-expression of Nef with Csk, the negative regulator of SFKs, also did not induce yeast protein-tyrosine phosphorylation (right panels). In contrast, expression of c-Src (in the absence of Csk) led to strong tyrosine phosphorylation of yeast proteins. These results indicate that expression of Nef alone or Nef with Csk does not induce tyrosine phosphorylation of yeast proteins, consistent with the lack of endogenous SFK orthologs in yeast. (TIFF) [file pone.0032561.s001.tiff]

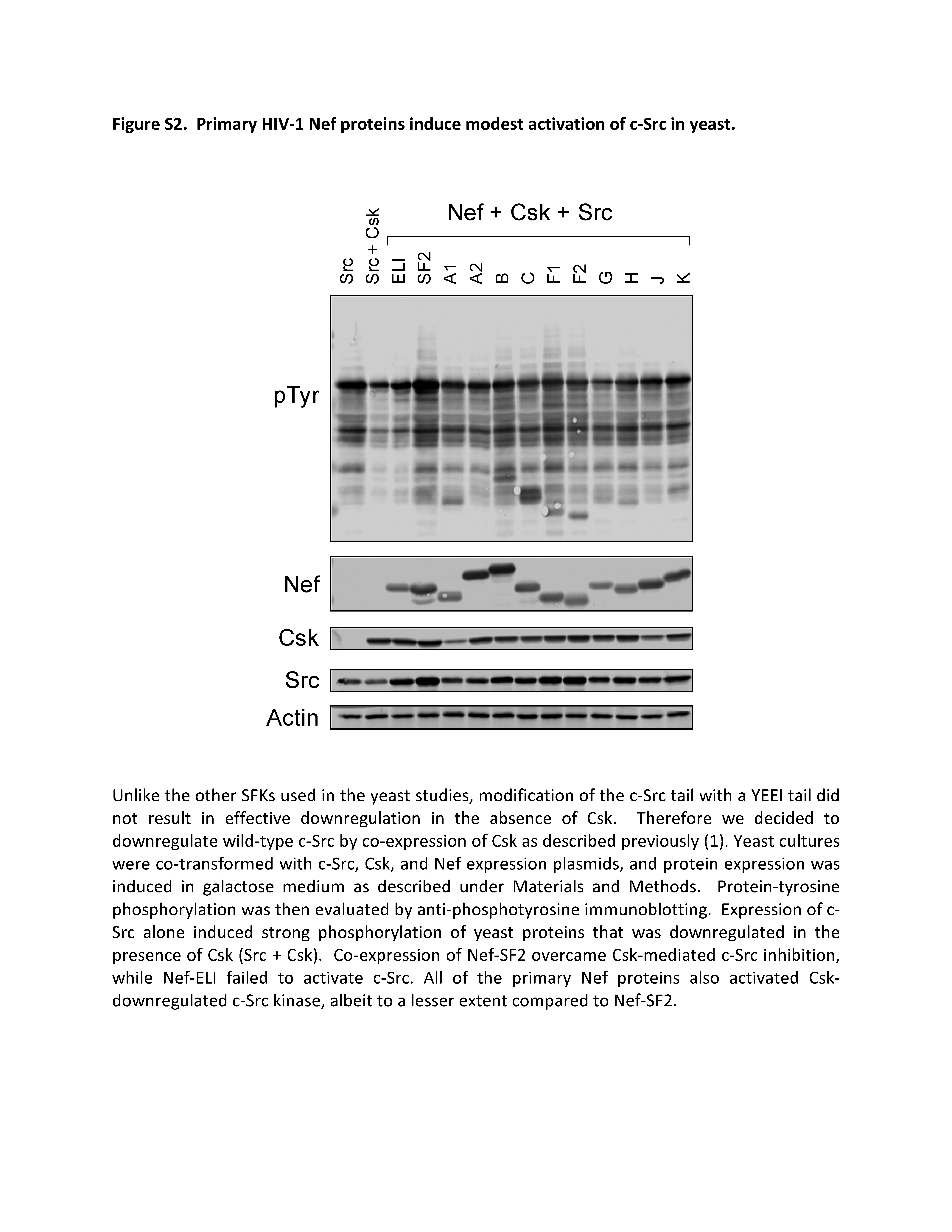

Supplement: Figure S2 — Primary HIV-1 Nef proteins induce modest activation of c-Src in yeast. Unlike the other SFKs used in the yeast studies, modification of the c-Src tail with a YEEI tail did not result in effective downregulation in the absence of Csk. Therefore we decided to downregulate wild-type c-Src by co-expression of Csk as described previously. Yeast cultures were co-transformed with c-Src, Csk, and Nef expression plasmids, and protein expression was induced in galactose medium as described under Materials and Methods. Protein-tyrosine phosphorylation was then evaluated by anti-phosphotyrosine immunoblotting. Expression of c-Src alone induced strong phosphorylation of yeast proteins that was downregulated in the presence of Csk (Src+Csk). Co-expression of Nef-SF2 overcame Csk-mediated c-Src inhibition, while Nef-ELI failed to activate c-Src. All of the primary Nef proteins also activated Csk-downregulated c-Src kinase, albeit to a lesser extent compared to Nef-SF2. (TIFF) [file pone.0032561.s002.tiff]

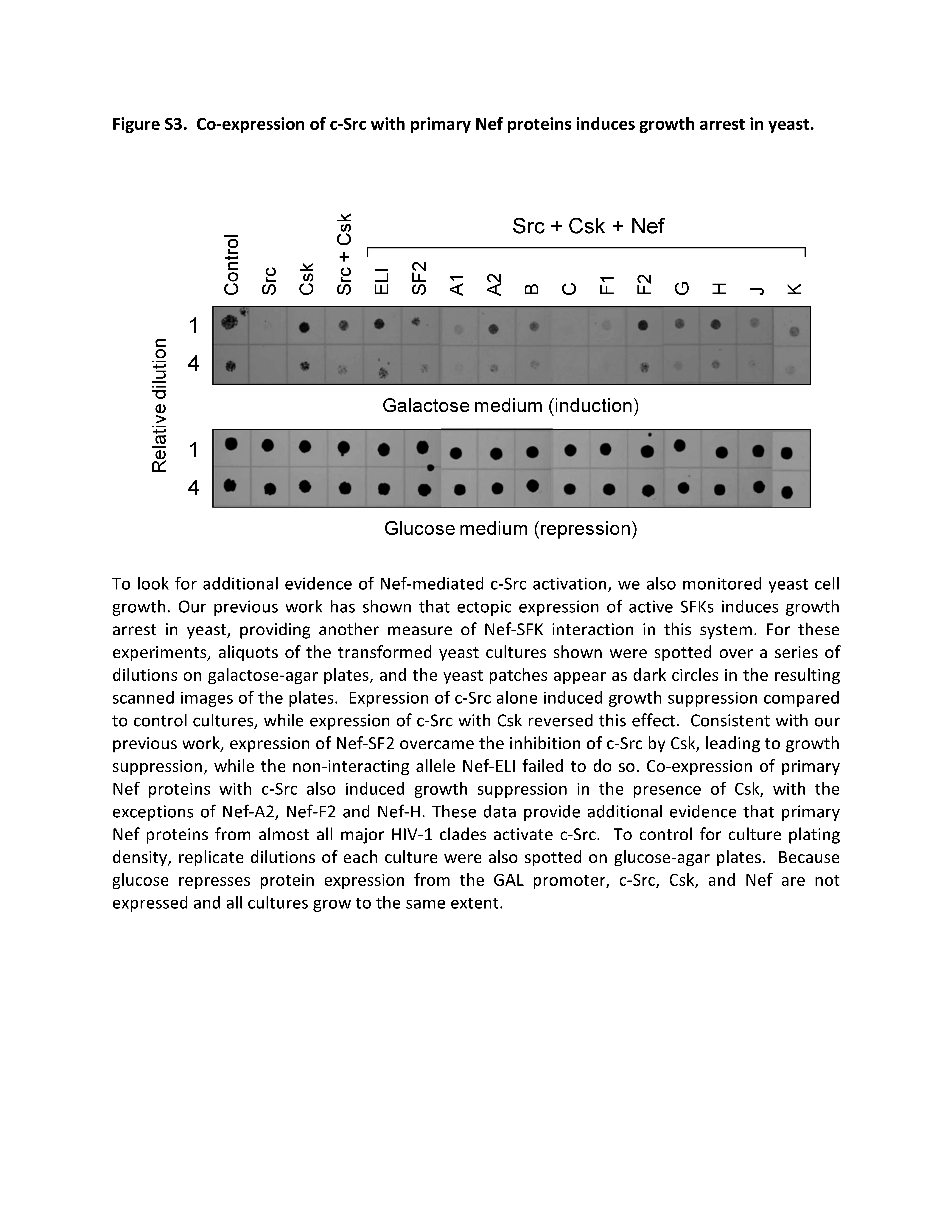

Supplement: Figure S3 — Co-expression of c-Src with primary Nef proteins induces growth arrest in yeast. To look for additional evidence of Nef-mediated c-Src activation, we also monitored yeast cell growth. Our previous work has shown that ectopic expression of active SFKs induces growth arrest in yeast, providing another measure of Nef-SFK interaction in this system. For these experiments, aliquots of the transformed yeast cultures shown were spotted over a series of dilutions on galactose-agar plates, and the yeast patches appear as dark circles in the resulting scanned images of the plates. Expression of c-Src alone induced growth suppression compared to control cultures, while expression of c-Src with Csk reversed this effect. Consistent with our previous work, expression of Nef-SF2 overcame the inhibition of c-Src by Csk, leading to growth suppression, while the non-interacting allele Nef-ELI failed to do so. Co-expression of primary Nef proteins with c-Src also induced growth suppression in the presence of Csk, with the exceptions of Nef-A2, Nef-F2 and Nef-H. These data provide additional evidence that primary Nef proteins from almost all major HIV-1 clades activate c-Src. To control for culture plating density, replicate dilutions of each culture were also spotted on glucose-agar plates. Because glucose represses protein expression from the GAL promoter, c-Src, Csk, and Nef are not expressed and all cultures grow to the same extent. (TIFF) [file pone.0032561.s003.tiff]

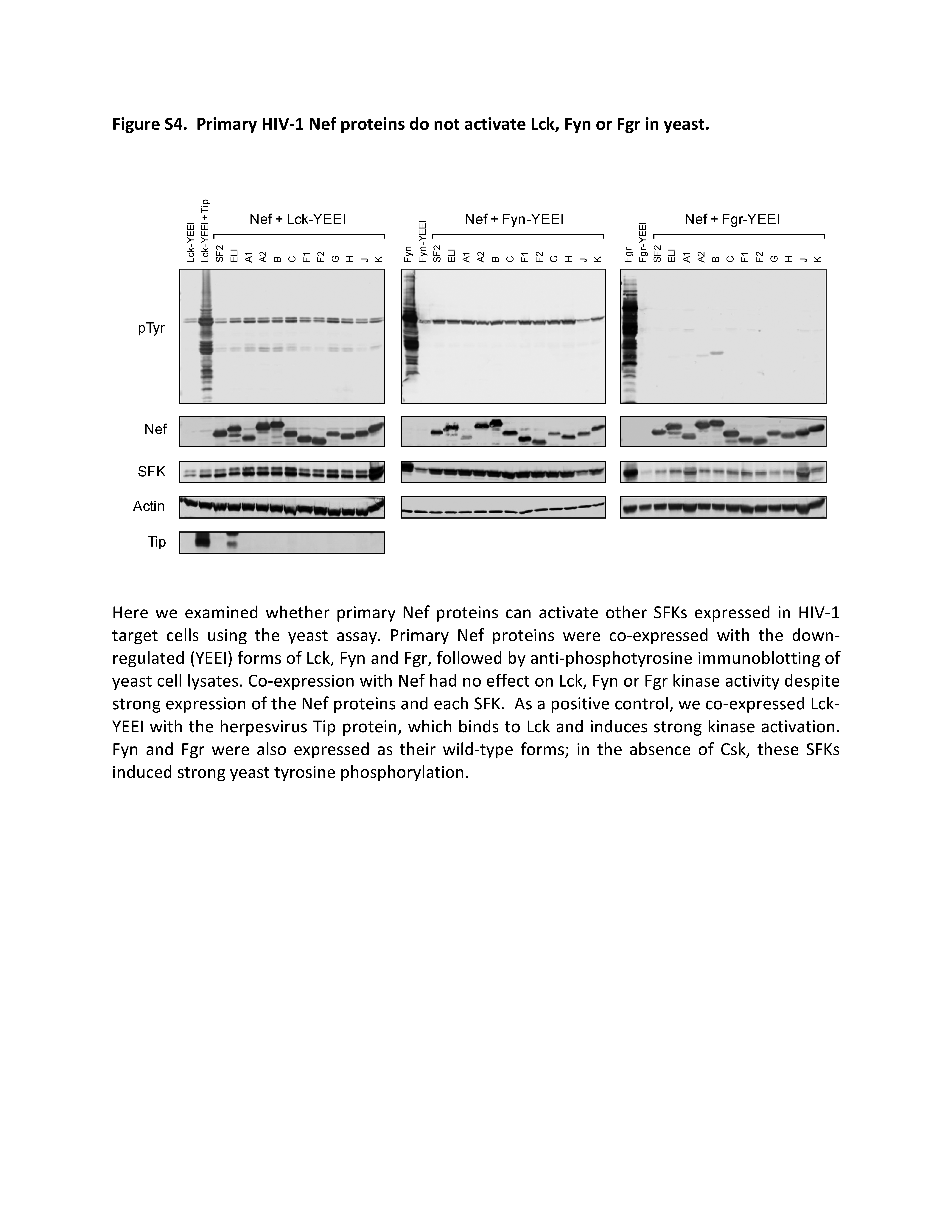

Supplement: Figure S4 — Primary HIV-1 Nef proteins do not activate Lck, Fyn or Fgr in yeast. Here we examined whether primary Nef proteins can activate other SFKs expressed in HIV-1 target cells using the yeast assay. Primary Nef proteins were co-expressed with the down-regulated (YEEI) forms of Lck, Fyn and Fgr, followed by anti-phosphotyrosine immunoblotting of yeast cell lysates. Co-expression with Nef had no effect on Lck, Fyn or Fgr kinase activity despite strong expression of the Nef proteins and each SFK. As a positive control, we co-expressed Lck-YEEI with the herpesvirus Tip protein, which binds to Lck and induces strong kinase activation. Fyn and Fgr were also expressed as their wild-type forms; in the absence of Csk, these SFKs induced strong yeast tyrosine phosphorylation. (TIFF) [file pone.0032561.s004.tiff]

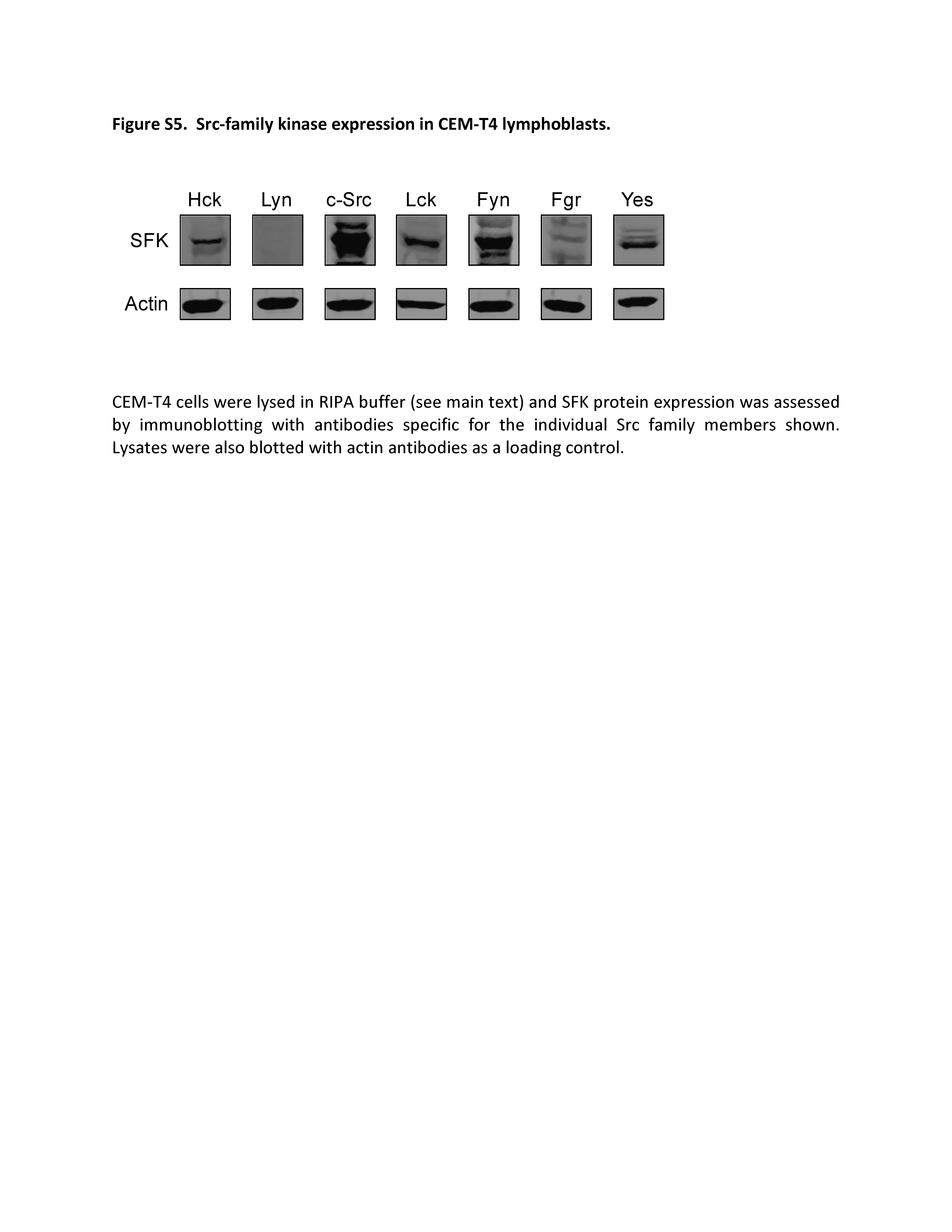

Supplement: Figure S5 — Src-family kinase expression in CEM-T4 lymphoblasts. CEM-T4 cells were lysed in RIPA buffer (see main text) and SFK protein expression was assessed by immunoblotting with antibodies specific for the individual Src family members shown. Lysates were also blotted with actin antibodies as a loading control. (TIFF) [file pone.0032561.s005.tiff]
